# Migration and preterm birth in war refugees: a Swedish cohort study

**DOI:** 10.1007/s10654-013-9877-9

**Published:** 2014-01-14

**Authors:** Can Liu, Marcelo Urquia, Sven Cnattingius, Anders Hjern

**Affiliations:** 1 Centre for Health Equity Studies (CHESS), Karolinska Institutet/Stockholm University, 106 91 Stockholm, Sweden; 2 Clinical Epidemiology Unit, Department of Medicine, Solna, Karolinska Institutet, 171 77 Stockholm, Sweden; 3 Centre for Research on Inner City Health, The Keenan Research Centre in the Li Ka Shing Knowledge Institute, St. Michael’s Hospital, Toronto, Canada; 4 Dalla Lana School of Public Health, University of Toronto, Toronto, Canada

The preterm birth rate varies greatly between different migrant populations in high income countries, but the reasons have not been well elucidated [1]. War refugees have high rates of mental health problems such as post-traumatic stress disorder (PTSD), depression and anxiety disorder, as a consequence of exposure to chronic stress associated with war in the country of origin [2]. Exposure to stress often continues during the asylum seeking process in the country of resettlement, as evidenced by an altered stress hormone profile [3]. Female refugees of child-bearing ages are more likely to be victims of traumatic events and also more vulnerable to such stress exposure [4]. We hypothesized that exposure to this “refugee stress” during pregnancy may lead to an increased risk of preterm birth and that this effect would fade with time after resettlement.

To investigate the association between duration of residence and preterm birth risk among war refugees, we exploited data from the Swedish national registers. We included information on all live singletons births between 1992 and 2008 of women during their first 5 years of residency in Sweden, who were at least 13 years of age when they had obtained Swedish residency and had immigrated from Yugoslavia (1992–2000), Iraq (1992–2004), Somalia (1992–2004), or Afghanistan (1992–2004) with a spouse of the same origin. Information on perinatal indicators and maternal age was retrieved from the Swedish Medical Birth Register (SMBR) and country of birth and year of immigration from the Register of the Total Population. Those with a birth weight for gestational age >3 standard deviations (SDs) or <−6 SDs according to the Marsal growth chart [5], were excluded as probable coding errors. The cut-off limits were based on the assumption that being very small for gestational age is more probable than being very large for gestational age in preterm infants. After also excluding infants with a malformation other than undescended testicle, preauricular appendage, congenital nevus or hip dislocation, the study population included 20,723 births.

Number of years since immigration were categorized into 0, 1, or 2–4 years. Information on maternal age, parity, mode of delivery, diagnoses during pregnancy and delivery, birth weight, gestational age, and sex of offspring were retrieved from SMBR. Diagnoses were coded according to the Swedish version of the International Classification of Diseases. The ninth revision (ICD-9) was used between 1987 and 1996, and the tenth revision (ICD-10) has been used thereafter. Gestational age was estimated by early second trimester ultrasound with reference to the Marsal growth chart [5]. If ultrasound estimation was not available, information on the last menstrual period was used to estimate gestational age. Preterm birth was defined as birth at 22–36 completed gestational weeks, and was further divided into very (22–32 weeks) and moderately preterm (33–36 weeks). Information on onset of labor was categorized into spontaneous onset of labor and induced labor, the latter including vaginally induced labor and planned cesarean delivery, based on the information routinely collected by the midwife at delivery. Births with overlapping records of spontaneous and induced labor were categorized as spontaneous onset of labor. All births with diagnosis of premature rupture of membranes (PPROM: ICD-9 code 658.1 and ICD-10 code O42) were further included as spontaneous onset of labor.

To estimate the association between duration of residence and preterm birth, a logistic regression model was used, adjusting for maternal country of origin, age, parity and sex of the infant. We further used multinomial logistic regression to estimate ORs for very preterm birth and moderately preterm birth, adjusting for the abovementioned covariates. All statistical analyses were performed using STATA version 12 for Windows. The study was approved by the regional ethics committee in Stockholm.

Compared with the second year of residency, the OR of preterm birth (<37 weeks) was 1.39 (95 % CI 1.13–1.72) in the first year of residency and 1.10 (95 % CI 0.94–1.28) in the third to fifth year of residency. Compared with the second year of residency, the ORs of very and moderately preterm birth in the first year of residence were 2.15 (95 % CI 1.37–3.38) and 1.23 (95 % CI 0.98–1.57), respectively (Table 1). Giving birth in the third to fifth year of residence was associated with an increased risk of very preterm birth (OR = 1.54 95 % CI 1.07–2.21) compared with the second year, but not of moderately preterm birth (OR = 1.01 95 % CI 0.85–1.20). Among all preterm births, 72.9 % had spontaneous onset of labor, 21.7 % had induced onset of labor, and 5.5 % had unknown onset of labor. The ORs of spontaneous preterm birth and induced preterm birth versus term birth were similar in the first year of residence compared with the second year, 1.40 (95 % CI 1.09–1.78) versus 1.27 (95 % CI 0.80–2.00), as well as in the third to fifth year, 1.09 (95 % CI 0.78–1.52) versus 1.08 (95 % CI 0.90–1.29.

**Table 1:** Binomial and multinomial logistic regression of the association between duration of residence and preterm birth and subtypes of preterm birth (N = 20,723)

Variables	N	Preterm		Very preterm		Moderately preterm	
		Incidence %	OR (95 % CI)	Incidence %	OR (95 % CI)	Incidence %	OR (95 % CI)
*Duration of residence (years)*							
1st	2,619	5.7	**1.39** (1.13–1.72)	1.5	**2.15** (1.37–3.38)	4.2	1.24 (0.98–1.57)
2nd	5,988	4.2	1	0.7	1	3.5	1
3rd–5th	12,116	4.5	1.10 (0.94–1.28)	1.0	**1.54** (1.07–2.21)	3.5	1.01 (0.85–1.20)
*Infant sex*							
Male	10,722	4.7	1	1.0	1	3.7	1
Female	10,001	4.3	0.92 (0.80–1.05)	0.9	0.90 (0.68–1.20)	3.4	0.92 (0.80–1.07)
*Maternal country of birth*							
Former Yugoslavia	8,658	4.8	1	1.1	1	3.7	1
Iraq	8,832	4.6	0.98 (0.84–1.13)	1.0	0.94 (0.69–1.27)	3.6	0.99 (0.84–1.16)
Somalia	2,398	3.4	**0.76** (0.60–0.98)	0.5	**0.54** (0.30–0.98)	2.9	0.82 (0.63–1.08)
Afghanistan	835	5.3	1.19 (0.86–1.64)	1.0	0.97 (0.47–2.04)	4.3	1.21 (0.87–1.78)
*Maternal age (years)*							
13–19	802	5.3	1.12 (0.80–1.56)	0.8	0.62 (0.27–1.43)	4.5	1.29 (0.90–1.85)
20–24	5,753	4.1	0.90 (0.77–1.07)	0.7	**0.62** (0.42–0.90)	3.4	0.99 (0.83–1.19)
25–34	11,750	4.3	1	1.0	1	3.3	1
35+	2,418	6.4	**1.59** (1.31–1.93)	1.6	0.97 (0.47–2.04)	4.8	**1.58** (1.27–1.97)
*Parity*							
0	7,259	4.9	1	1.1	1	3.8	1
1–2	10,952	4.3	0.87 (0.74–1.01)	0.8	**0.65** (0.47–0.90)	3.5	0.94 (0.79–1.12)
3+	2,512	4.5	0.79 (0.61–1.00)	1.2	0.79 (0.48–1.27)	3.3	0.78 (0.59–1.04)

Bold values indicate *P* < 0.05

This study demonstrates that war refugees in Sweden have an increased risk of preterm birth, especially very preterm birth, during their first year of residency compared with the following year. In congruence with our hypothesis, our results suggest that war- and migration-related stress may cause an increased risk of preterm birth of short duration.

Post-traumatic stress symptoms, which are very common in war refugees, are consistently associated with alterations in the hypothalamic–pituitary–adrenal (HPA) axis. High levels of corticotrophin-releasing hormone (CRH) are very common, while cortisol levels vary much more, depending on the duration of stress [6]. High plasma levels of CRH in the second trimester, but not high levels of cortisol, are associated with increased risks of preterm birth [7]. Studies of self-reported stress have not been able to link subjective stress with preterm birth, while bereavement, another natural experiment of stress, has shown a slight association with preterm birth in large population based studies [8]. Major alterations in the HPA-axis are not linked to normal bereavement reactions, but are consistently found in major depression, which can be triggered by bereavement in vulnerable individuals [9]. Thus, further studies of stress and preterm birth need to pay particular attention to the HPA-axis where CRH seems to be a key indicator.

We recognize that stress is not the only possible explanation for the higher risk of preterm birth in our study population. One may hypothesize that other exposures in the country of origin, such as malnutrition and infectious agents may also be involved, as well as informal barriers to care in the resettlement country.

We also found an increased risk of very preterm but not moderately preterm birth in the third to fifth year of residency. This confirms a time pattern after migration that has previously been demonstrated in immigrants in Canada in a longer time scale [10], although the reason for this increase is yet to be explained. One may speculate that other risk factors could emerge after a longer period of residence in Sweden, e.g. unemployment and acculturation to risky health behaviors and diets. Further studies are needed to elucidate the mechanisms for these time dependent patterns of preterm birth for migrants.

Our study is limited by the lack of precise information on the timing of migration in our study population; we lacked information on when the refugees set foot on Swedish soil, and the exact date of when residency was acquired. Thus, our estimates probably underestimate the time dependency of preterm birth in relation to migration. The sample size also restricted further investigation of potential mechanisms of preterm birth.

In conclusion, this study demonstrates an increased risk of preterm birth in war refugees during the first year of residency in Sweden. This suggests that different levels of stress, including post-traumatic stress, might explain some of the differences in rates of preterm birth between migrant populations. Further studies with more specific measures of stress and hormonal profiles are needed to investigate stress as a risk factor for preterm birth in migrant populations.
